# Distributed Robust Filtering for Wireless Sensor Networks with Markov Switching Topologies and Deception Attacks

**DOI:** 10.3390/s20071948

**Published:** 2020-03-31

**Authors:** Fengzeng Zhu, Xu Liu, Jiwei Wen, Linbo Xie, Li Peng

**Affiliations:** 1 Engineering Research Center of Internet of Things Applied Technology, Jiangnan University, Ministry of Education, Wuxi 214122, Jiangsu, China; zhufengzeng@stu.jiangnan.edu.cn (F.Z.);; 2 Jiangsu Province Internet of Things Application Technology Key Construction Laboratory, Wuxi Taihu College, Wuxi 214063, Jiangsu, China

**Keywords:** wireless sensor network, switching topology, deception attack, distributed filtering, linear matrix inequality

## Abstract

This paper is concerned with the distributed full- and reduced-order $l_{2}$-$l_{\infty }$ state estimation issue for a class of discrete time-invariant systems subjected to both randomly occurring switching topologies and deception attacks over wireless sensor networks. Firstly, a switching topology model is proposed which uses homogeneous Markov chain to reflect the change of filtering networks communication modes. Then, the sector-bound deception attacks among the communication channels are taken into consideration, which could better characterize the filtering network communication security. Additionally, a random variable obeying the Bernoulli distribution is used to describe the phenomenon of the randomly occurring deception attacks. Furthermore, through an adjustable parameter *E*, we can obtain full- and reduced-order $l_{2}$-$l_{\infty }$ state estimator over sensor networks, respectively. Sufficient conditions are established for the solvability of the addressed switching topology-dependent distributed filtering design in terms of certain convex optimization problem. The purpose of solving the problem is to design a distributed full- and reduced-order filter such that, in the presence of deception attacks, stochastic external interference and switching topologies, the resulting filtering dynamic system is exponentially mean-square stable with prescribed $l_{2}$-$l_{\infty }$ performance index. Finally, a simulation example is provided to show the effectiveness and flexibility of the designed approach.

## 1. Introduction

In the past few decades, wireless sensor networks (WSNs) have received widespread attention due to their enormous application potential in military installations, environmental monitoring, information collection, and power grids, see [1,2,3,4,5,6,7,8,9,10]. Unlike a single sensor in a traditional system, a sensor node in WSNs can collaborate with other sensors in a given topology based on information measured by itself and output information of adjacent sensors. Therefore, some initiatives have been taken to address the problems of distributed filtering based on WSNs in recent years, see [11,12,13,14,15,16,17,18,19,20]. For example, in [12], the filtering problem of nonlinear stochastic systems affected by sensor saturation in unstable communication channels has been solved. By using standard Kalman filter updating and consistency updating, a two-stage Kalman consistency filtering method has been proposed in [14]. Additionally in [18], the distributed robust filtering problem has been solved for a class of discrete T-S fuzzy systems with immeasurable premise variables and Sigma-Delta quantiser over WSNs. Besides, a distributed non-linear Kalman filter with a more general communication scheme has been presented in [19], which processed the additive white Gaussian noise in the system, measurement and communication. Furthermore, the developed algorithm has been applied to estimate the unknown information distribution in a certain area of the optimal coverage control problem over sensor networks. Based on the piecewise Lyapunov function approach and the concept of average dwell time, a convex optimization method has been used to deal with the distributed $H_{\infty }$ filtering problem for a stochastic switching system with a fixed delay and a fading measurement over the sensor network in [20].

One of the important requirements of those applications over WSNs is high level of security while transferring information. Deception attacks, denial of service attacks and false information injection attacks are the mostly frequently occurred phenomena over WSNs. These attacks should be adequately considered during system analysis/synthesis to prevent undesired behavior (e.g., degradation, divergence, and instability), thus have attracted considerable attention during the past few years, see [21,22,23]. In addition, in practical applications, many factors may cause random changes in the topology of communication networks. For example, instability of communication link between filters, obstacle blocking, network-induced factors, adding new nodes due to application requirements, etc. In recent years, many achievements have been made on this issue, see [24,25,26,27]. As such, a seemingly natural question is that, when the WSNs are subject to both switching topologies and deception attacks, we need to design the distributed robust filter to realize system state estimation.

### 1.1. Related Work

There are several techniques including linear matrix inequality (LMI) approach, parameter-dependent/recursive LMI method, and Riccati difference equation method for distributed filtering or estimation problems, see [28,29,30,31,32,33]. For example, by using the LMI technology, a desired sampled data-based distributed filter has been constructed in [32] for stochastic nonlinear systems over WSNs with multiple bounded time-delays, the distributed filtering problem has been investigated in [33] for a class of discrete-time stochastic systems over WSNs with randomly missing measurements and varying nonlinearities, and the distributed state estimators have been designed in [28,31] for nonlinear systems with multiple network-induced phenomena. Besides, in order to reflect a more realistic situation, in [29,30], randomly packet dropouts, randomly occurring uncertainties and nonlinearities have been considered during designing event-triggered distributed filters.

More recently, surveys on deception attacks began to appear, see [34,35,36,37,38,39]. For instance, in [34], the most general model of sensor attack resilient has been proposed, which can allow any signal to be injected through the compromised sensor. Based on this model, the attack resilient state estimation problem has been studied under bounded noise. The effect of stealthy integrity attacks on Networked physical systems has been analyzed in [36], where the target system was a Stochastic Linear Time-Invariant (LTI) system with a linear estimator, a linear feedback controller and a $\chi^{2}$ fault detector. In [35,37,38], the distributed state estimator, which was used to defend against false data injection attacks over sensor networks, has been embedded with event-triggering transmission scheme. Based on clustering technology, which can remove bad data and/or inaccurate estimates, a distributed Kalman filtering approach with trust-based dynamic combination strategy has been proposed in [39] to enhance the robustness against cyber attacks.

The corresponding research on switching topologies has been received preliminary attention due to its engineering insights, see [13,40,41,42,43,44,45,46]. For example, in [40], the distributed filtering problem of network topology with limited switching frequency has been investigated. In [41,42], a novel distributed $H_{\infty }$ state estimation framework for discrete-time partial information exchange has been proposed by modeling time-varying network topologies with non-homogeneous Markov chains. In [43,44,45], the designed distributed robust filtering has been considered both Markov switching communication topologies and event triggering mechanism. Considering the two types of network-induced communication constraints that occur during sensor measurement and transmission between filters, i.e., channel fading and random packet dropouts, the distributed $H_{\infty }$-consensus problem with communication topology switching has been solved in [46] under those constraints. Up to now, the stability analysis and control, filtering and fault detection of Markov jump systems have been investigated in [47,48,49,50,51].

### 1.2. Main Contribution

It should be noted that, most existing design methods have been focused on distributed full-order filering, while little effort has been devoted to design distributed reduced-order filering, not to mention that both types of filters can be obtained simultaneously. As we all know, the distributed reduced-order filters can reduce the complexity of the filtering network and facilitate engineering application. In order to defend the effects of randomly deception attacks, switching communication topologies and external interference for WSNs, the design of distributed *l*-order robust filters is important and challenging. However, the current research results are not abundant in the *l*-order distributed state estimation over WSNs subjected to randomly deception attacks and switching communication topologies. Therefore, we aim to design a distributed full- and reduced-order filtering algorithm for a class of discrete time-invariant systems over WSNs, all possible unknown disturbances, deception attacks and switching topologies, the following design objectives are achieved simultaneously: (i) the distributed filtering dynamic system is exponentially stable in the mean square sense and (ii) a prescribed $l_{2}$-$l_{\infty }$ performance index is guaranteed.

The main contributions of this paper are summarized as follows:

(1) Through an adjustable parameter *E*, we can obtain full- and reduced-order $l_{2}$-$l_{\infty }$ state estimationer over sensor networks, respectively;

(2) The sector-bound deception attacks among the communication channels are taken into consideration, which could better characterize the filtering network communication security;

(3) Sufficient conditions are established for the solvability of the addressed switching topology-dependent distributed filtering design in terms of certain convex optimization problem.

The remainder of this paper is organized as follows: In Section 2, the studied issues and basic definitions are formulated. In Section 3 and Section 4 mainly introduces the performance analysis and the *l*-order filter design method, respectively. The effectiveness and flexibility of designed distributed filter is verified in Section 5. Finally, we conclude this paper in Section 6.

**Notation.** In this paper, $R^{n}$ and $R^{n\times m}$ denote the *n* dimensional Euclidean space and the set of n×m all real matrices, respectively. ∥·∥ refers to the Euclidean norm. The notation X>0 means that matrix *X* is real positive definite symmetric matrix. The superscript *T* stands for matrix transposition. *I* denotes the identity matrix of compatible dimension. $diag_{n}\left\{A_{i}\right\}$ stands for the block-diagonal matrix $diag\left\{A_{1},A_{2},\ldots ,A_{n}\right\}$, and $diag_{n}\left\{A\right\}$ means the *N*-block-diagonal matrix $diag\left\{A,A,\cdots ,A\right\}$. $diag_{n}^{i}\left\{A\right\}$ represents the *N*-bolck-diagonal matrix with its *i*-th block *A* and the others zero matrices. $vec_{n}\left\{x_{i}\right\}$ denotes row vector $\left[x_{1},x_{2},\cdots ,x_{n}\right]$. E{x} stands for the mathematical expectation of the stochastic variable *x*. The symbol “∗” describes the symmetric part in a symmetric block matrix.

## 2. Problem Statement

### 2.1. Preliminaries

Consider a G = $\left(V,E,A\right)$ sensor network whose topology is represented by a given digraph G of order *n* with the set of nodes V = {1,2,⋯,n}, set of edges E⊆V×V, and a weighted adjacency matrix $A={\left[a_{ij}\right]}_{n\times n}\left(a_{ij}\geq 0\right)$ with nonnegative adjacency elements $a_{ij}$. An edge of G is denoted by the ordered pair (i,j). The adjacency elements associated with the edges of the graph are positive, i.e., $a_{ij}>0$. Also, we assume $a_{ii}=1$ for all i∈V, and therefore (i,i) can be regarded as an additional edge. The set of neighbors of node i∈V plus the node itself are denoted by $N_{i}=\left\{j\in V:\left(i,j\right)\in E\right\}$.

### 2.2. System Model

In this paper, the target plant is a system whose state is estimated by distributed sensors. Consider the following discrete-time linear networked system:

(1) $$\left\{\begin{array}{c} \begin{array}{ccc} x(k+1) & = & Ax\left(k\right)+Bw\left(k\right),x\left(0\right)=x_{0}\\ z(k) & = & Mx(k) \end{array} \end{array}\right.$$

where $x\left(k\right)\in R^{n_{x}}$ is the system state vector, $z\left(k\right)\in R^{n_{z}}$ the signal to be estimated, and $w\left(k\right)\in R^{n_{w}}$ is the exogenous disturbance input that belongs to $l_{2}\left[0,\infty \right)$. A,B, and *M* in system (1) are known matrices with proper dimensions.

**Figure 1:**
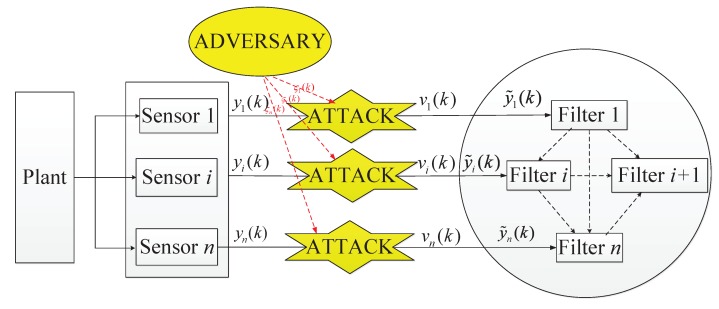
Distributed filtering network with deception attacks.

As shown in Figure 1, this paper studies the design of dynamic target state estimation in sensor networks under random attacks. The considered attack type is deception information injection. The attack strategy is to randomly inject malicious vectors into the transmitted measurements of sensor nodes via wireless channel between sensor network and filtering network.

Consider the following sensor measurement models and attack models:

(2) $$\left\{\begin{array}{c} \begin{array}{ccc} y_{i}\left(k\right) & = & C_{i}x\left(k\right)\\ {\tilde{y}}_{i}\left(k\right) & = & y_{i}\left(k\right)+\theta_{i}\left(k\right)\varsigma_{i}\left(k\right)+D_{i}v_{i}\left(k\right)\\ \varsigma_{i}\left(k\right) & = & -y_{i}\left(k\right)+\phi_{i}\left(y_{i}\left(k\right)\right) \end{array} \end{array}\right.$$

where $y_{i}\left(k\right)\in R^{n_{y}}$ is the measurement output received by sensor *i* from system (1). ${\tilde{y}}_{i}\left(k\right)$ is the transmitted measurement values via the network subject to deception attacks, the signal $\varsigma_{i}\left(k\right)$, $v_{i}\left(k\right)$ is the random channel noise belonging to $l_{2}\left[0,\infty \right)$, and $\phi_{i}\left(y_{i}\left(k\right)\right)$ is a malicious signal injected by the attacker, which is inaccessible to the defenders. $C_{i}$ and $D_{i}$ are known matrices with proper dimensions. In addition, we assume that $\phi_{i}\left(y_{i}\left(k\right)\right)$ satisfies the nonlinear vector-valued function $\phi_{i}\left(\cdot \right):R^{n_{\tilde{y}}}\to R^{n_{\tilde{y}}}$.

The nonlinear function $\phi_{i}\left(\cdot \right)$ is continuous and bounded, and satisfies the following inequality:

(3) $${\left[\phi_{i}\left(y_{i}\left(k\right)\right)-K_{1}y_{i}\left(k\right)\right]}^{T}\left[\phi_{i}\left(y_{i}\left(k\right)\right)-K_{2}y_{i}\left(k\right)\right]\leq 0$$

for all ${\tilde{y}}_{i}\left(k\right)\in R^{n_{\tilde{y}}}$, where $K_{1}$ and $K_{2}$ are real matrices with appropriate dimensions.

The random variable $\theta_{i}\left(k\right)$ is Bernoulli distributed white sequence, mutually independent and not equired to be equal to each other, satisfying the following probability distribution:

(4) $$\begin{array}{ccc} Prob\left\{\theta_{i}\left(k\right)=1\right\} & = & E\left\{\theta_{i}\left(k\right)\right\}=\beta_{i},\;Prob\left\{\theta_{i}\left(k\right)=0\right\}=E\left\{1-\theta_{i}\left(k\right)\right\}=1-\beta_{i},\\ E\left\{{\left(\theta_{i}\left(k\right)-\beta_{i}\right)}^{2}\right\} & = & \alpha_{i},\;E\left\{\left(\theta_{i}\left(k\right)-\beta_{i}\right)\left(\theta_{j}\left(k\right)-\beta_{j}\right)\right\}=0,\left(i\neq j\right), \end{array}$$

where $\beta_{i}$ is a known nonnegative constant.

Due to the topology of the sensor networks, for each sensor node, the filter to be designed is assumed to be of the following form:

(5) $$\left\{\begin{array}{c} \begin{array}{cc} {\hat{x}}_{i}\left(k+1\right) & =\sum_{j\in N_{{}_{i}}^{r(k)}}a_{ij}^{r(k)}W_{{}_{ij}}^{r(k)}{\hat{x}}_{j}\left(k\right)+\sum_{j\in N_{{}_{i}}^{r(k)}}a_{ij}^{r(k)}H_{{}_{ij}}^{r(k)}{\tilde{y}}_{j}\left(k\right)\\ {\hat{z}}_{i}\left(k\right) & =L_{i}^{r(k)}{\hat{x}}_{i}\left(k\right) \end{array} \end{array}\right.$$

where ${\hat{x}}_{i}\in R^{l}$ is the filter state, ${\hat{z}}_{i}\left(k\right)\in R^{n_{z}}$ is the estimated output. In addition, we assume that *l* represents the order of the filter. If l=m, the filter belongs to a full-order filter. Accordingly, it is a reduced-order filter when 1≤l<m.

The Markov chain r(·) take values in a finite set $S=\{1,2,\cdots ,n_{0}\}$, $\Pi ={\left[\pi_{st}\right]}_{n_{0}\times n_{0}}$ represents the probability of jumping from topology *s* to topology *t*, which can be expressed as:

(6) $$\pi_{st}=Prob\left(r\left(k+1\right)=t|r\left(k\right)=s\right).\;\forall s,t\in S$$

where $\pi_{st}\geq 0$, $\sum_{t=1}^{n_{0}}\pi_{st}=1$. Here, matrices $W_{{}_{ij}}^{r(k)}$, $H_{{}_{ij}}^{r(k)}$, $L_{i}^{r(k)}\left(j\in N_{i}^{r(k)}\right)$ in (5) are filtering parameters to be determined.

### 2.3. Augmented Filtering Error System Model

For the sake of convenience, we define

(7) $$\begin{array}{ccc} \hat{x}\left(k\right) & = & vec_{n}^{T}\left\{{\hat{x}}_{i}^{T}\left(k\right)\right\},\;\bar{x}\left(k\right)=vec_{n}^{T}\left\{x^{T}\left(k\right)\right\},\;\phi \left(y\left(k\right)\right)=vec_{n}^{T}\left\{\phi_{i}^{T}\left(y_{i}\left(k\right)\right)\right\},\\ v(k) & = & vec_{n}^{T}\left\{v_{i}^{T}\left(k\right)\right\},\;e\left(k\right)=vec_{n}^{T}\left\{e_{i}^{T}\left(k\right)\right\},\;\tilde{y}\left(k\right)=vec_{n}^{T}\left\{{\tilde{y}}_{i}^{T}\left(k\right)\right\},\\ \bar{B} & = & vec^{T}\left\{B^{T}\right\},\;\bar{A}=diag_{n}\left\{A\right\},\;\bar{M}=diag_{n}\left\{M\right\},\;\bar{D}=diag_{n}\left\{D_{i}\right\},\\ {\bar{C}}_{1-\beta } & = & diag_{n}\left\{\left(1-\beta_{i}\right)C_{i}\right\},\;{\bar{L}}^{r(k)}=diag_{n}\left\{L_{i}^{r(k)}\right\},\;\tilde{C}=\left[\bar{C}\;0\right],\\ {\bar{C}}_{n}^{i} & = & diag_{n}^{i}\left\{C_{i}\right\},\;{\bar{K}}_{1}=diag_{n}\left\{K_{1}\right\},\;{\bar{K}}_{2}=diag_{n}\left\{K_{2}\right\},\\ {\bar{E}}_{n}^{i} & = & diag_{n}^{i}\left\{I\right\},\;\bar{C}=diag_{n}\left\{C_{i}\right\},\;\alpha_{i}=\beta_{i}\left(1-\beta_{i}\right),\;\bar{\beta }=diag_{n}\left\{\beta_{i}\right\}. \end{array}$$

Defining $\eta \left(k\right)={\left[{\bar{x}}^{T}\;{\hat{x}}^{T}\right]}^{T}$, $\bar{w}\left(k\right)={\left[w^{T}\left(k\right)\;v^{T}\left(k\right)\right]}^{T}$, $e\left(k\right)=z\left(k\right)-\hat{z}\left(k\right)$. Then, it is easy to obtain the augmented filtering error system as follows:

(8) $$\begin{array}{c} \left\{\begin{array}{ccc} \eta (k+1) & = & \left[A^{s}+\sum_{i=1}^{n}\left(\theta_{i}\left(k\right)-\beta_{i}\right)F_{1i}^{s}\right]\eta \left(k\right)+\left[B_{1}^{s}+\sum_{i=1}^{n}\left(\theta_{i}\left(k\right)-\beta_{i}\right)F_{2i}^{s}\right]\phi \left(y\left(k\right)\right)+B_{2}^{s}\bar{w}\left(k\right)\\ e(k) & = & M^{s}\eta \left(k\right) \end{array}\right. \end{array}$$

where

(9) $$\begin{array}{cc} A^{s} & =\left[\begin{array}{cc} \bar{A} & 0\\ {\bar{H}}^{s}{\bar{C}}_{1-\beta } & {\bar{W}}^{s} \end{array}\right],\;F_{1i}^{s}=\left[\begin{array}{cc} 0 & 0\\ -{\bar{H}}^{s}{\bar{C}}_{n}^{i} & 0 \end{array}\right],\;F_{2i}^{s}=\left[\begin{array}{c} 0\\ {\bar{H}}^{s}{\bar{E}}_{n}^{i} \end{array}\right],\\ B_{1}^{s} & =\left[\begin{array}{c} 0\\ {\bar{H}}^{s}\bar{\beta } \end{array}\right],\;B_{2}^{s}=\left[\begin{array}{cc} \bar{B} & 0\\ 0 & {\bar{H}}^{s}\bar{D} \end{array}\right],\;M^{s}=\left[\begin{array}{cc} \bar{M} & -{\bar{L}}^{s} \end{array}\right], \end{array}$$

with

(10) $$\begin{array}{c} {\bar{W}}^{s}={\left[O_{ij}\right]}_{{}_{n\times n}}^{s},\;O_{ij}=a_{{}_{ij}}^{s}W_{ij}^{s},\\ {\bar{H}}^{s}={\left[{\bar{O}}_{ij}\right]}_{{}_{n\times n}}^{s},\;{\bar{O}}_{ij}=a_{{}_{ij}}^{s}H_{ij}^{s}. \end{array}$$

Clearly, since $a_{ij}=0$ when j∉$N_{i}^{s}$, ${\bar{W}}^{s}$ and ${\bar{H}}^{s}$ are two sparse matrices which can be described as:

(11) $${\bar{W}}^{s}\in W_{n_{x}\times n_{x}},\;{\bar{H}}^{s}\in W_{n_{x}\times n_{y}}$$

where

(12) $$W_{p\times q}=\left\{U=\left[U_{ij}\right]R^{np\times nq}|U_{ij}\in R^{p\times q},U_{ij}=0,if\;\;j\notin \;N_{i}^{s}\right\}.$$

Furthermore, it follows from (3) that

(13) $${\left[\phi \left(y\left(k\right)\right)-{\bar{K}}_{1}\tilde{C}\eta \left(k\right)\right]}^{T}\left[\phi \left(y\left(k\right)\right)-{\bar{K}}_{2}\tilde{C}\eta \left(k\right)\right]<0.$$

**Definition** **1.** *For a given disturbance attenuation level γ>0, the estimation error e(k) from (8) is said to satisfy the $l_{2}$-$l_{\infty }$ performance constraint if the following inequalities (a) and (b) holds simultaneously:*

*(a) For all η(0), the augmented system (8) with $\bar{w}\left(k\right)=0$ is exponentially mean-square stable if there exist scalars λ≥1 and 0<τ<1 such that*

(14) $$\left\{∥\eta (k)∥\right\}\leq \lambda \tau^{k}\left\{∥\eta (0)∥\right\}.$$

*(b) Under the zero-initial condition, for all nonzero $\bar{w}\left(k\right)$, the filter error e(k) satisfies*

(15) $$E\left\{{∥e(k)∥}_{\infty }^{2}\right\}<\gamma^{2}{∥\bar{w}\left(k\right)∥}_{2}^{2}$$

*where*

(16) $$\begin{array}{c} {∥e(k)∥}_{\infty }^{2}=\underset{k}{\sup}\left\{e^{T}\left(k\right)e\left(k\right)\right\},\\ {∥\bar{w}\left(k\right)∥}_{2}^{2}=\sum_{k=0}^{\infty }{\bar{w}}^{T}\left(k\right)\bar{w}\left(k\right). \end{array}$$

**Remark** **1.** *The $l_{2}$-$l_{\infty }$ performance index used in this paper is the constraint of the total output estimation error peak of all filter nodes at a certain time k for the whole filter network, which is less than the constraint of the estimated error peak of each filter node output.*

**Lemma** **1.** *(See the work of Yang et al. [52]) Assume that $V\left(\eta \left(k\right)\right)=\eta^{T}\left(k\right)P\eta \left(k\right)$ is a Lyapunov function. If there are scalars λ≥0,μ>0,v>0 and 0<ψ<1, such that*

(17) $$\mu {∥\eta (k)∥}^{2}\leq V\left(\eta \left(k\right)\right)\leq v{∥\eta (k)∥}^{2}$$

*and*

(18) $$E\left\{V(\eta (k+1)|\eta (k)\right\}-V\left(\eta \left(k\right)\right)\leq \lambda -\psi V\left(\eta \left(k\right)\right)$$

*then*

(19) $$E\left\{{∥\eta (k)∥}^{2}\right\}\leq \frac{v}{\mu }{\left(1-\psi \right)}^{k}E\left\{{∥\eta (0)∥}^{2}\right\}+\frac{\lambda }{\mu \psi }.$$

**Lemma** **2.** *(See the work of De et al. [53]) For matrices $A,Q=Q^{T}$ and P>0, the following matrix inequality*

(20) $$A^{T}PA-Q<0$$

*holds if and only if there exists a matrix T of appropriate dimensions such that*

(21) $$\left[\begin{array}{cc} -Q & *\\ T^{T}A & P-T-T^{T} \end{array}\right]<0.$$

**Lemma** **3.** *(See the work of Yang et al. [16]) Let $Q=diag\{Q_{1},Q_{2},\cdots ,Q_{n}\}$ with $Q_{i}\in R^{p\times p}\left(1\leq i\leq n\right)$ being invertible matrices. For $W\in R^{np\times nq}$ if X=QW, then we can obtain $W\in W_{p\times q}\Leftrightarrow X\in W_{p\times q}.$*

## 3. Distributed Filtering Performance Analysis

In this section, the stochastic stability condition in the mean square sense for augmented system (8) with switching topologies and deception attacks will be discussed, and the $l_{2}$-$l_{\infty }$ performance index is achieved.

**Theorem** **1.** *For given scalars γ>0. Assume that there exist matrices ${\bar{L}}^{s}=diag_{n}\left\{L_{i}^{s}\right\}$, P(s)>0, and two families of matrices ${\bar{W}}^{s}$, ${\bar{H}}^{s}$ satisfying the constraints*

(22) $${\bar{W}}^{s}\in W_{n_{x}\times n_{x}},{\bar{H}}^{s}\in W_{n_{x}\times n_{y}},s=1,2,\cdots ,n_{0}$$

*and the following set of LMIs*

(23) $$\left[\begin{array}{ccccc} \Gamma_{11}^{s} & * & * & * & *\\ \Gamma_{21}^{s} & -I & * & * & *\\ 0 & 0 & -I & * & *\\ \Gamma_{41}^{s} & \Gamma_{42}^{s} & \Gamma_{43}^{s} & \Gamma_{44}^{s} & *\\ \Gamma_{51}^{s} & \Gamma_{52}^{s} & 0 & 0 & \Gamma_{55}^{s} \end{array}\right]<0$$

(24) $$\left[\begin{array}{cc} -P(s) & *\\ M^{s} & -\gamma^{2}I \end{array}\right]<0$$

*where*

(25) $$\begin{array}{ccc} \Gamma_{11}^{s} & = & -P\left(s\right)-{\tilde{C}}^{T}{\bar{K}}_{1}^{T}{\bar{K}}_{2}\tilde{C},\;\Gamma_{21}^{s}=0.5\left({\bar{K}}_{1}+{\bar{K}}_{2}\right)\tilde{C},\\ \Gamma_{41}^{s} & = & \bar{P}\left(s\right)A^{s},\;\Gamma_{42}^{s}=\bar{P}\left(s\right)B_{1}^{s},\;\Gamma_{43}^{s}=\bar{P}\left(s\right)B_{2}^{s},\;\Gamma_{44}^{s}=-\bar{P}\left(s\right),\\ \Gamma_{51}^{s} & = & vec_{n}\left\{\sqrt{\alpha_{i}}\bar{P}\left(s\right)F_{1i}^{s}\right\},\;\Gamma_{52}^{s}=vec_{n}\left\{\sqrt{\alpha_{i}}\bar{P}\left(s\right)F_{2i}^{s}\right\},\;\Gamma_{55}^{s}=diag_{n}\left\{-\bar{P}\left(s\right)\right\}. \end{array}$$

**Proof** **of Theorem 1.** In order to prove the stability of the augmented system (8) (with $\bar{w}\left(k\right)=0$), we choose the Lyapunov function candidate as follows:

(26) $$V\left(\eta \left(k\right),s\right)=\eta^{T}\left(k\right)P\left(s\right)\eta \left(k\right).$$

Defining $\bar{P}\left(s\right)=\sum_{t=1}^{n_{0}}\pi_{st}P\left(t\right)$ and recalling the facts of (4), one has

(27) $$\begin{array}{cc} \Delta V(\eta (k),s) & =E\left\{V(\eta (k+1),t)|\eta (k),s\right\}-V\left(\eta \left(k\right),s\right)\\ \; & =E\left\{\eta^{T}\left(k+1\right)P\left(t\right)\eta \left(k+1\right)\right\}-\eta^{T}\left(k\right)P\left(s\right)\eta \left(k\right)\\ \; & =\eta^{T}\left(k\right){A^{s}}^{T}\bar{P}\left(s\right)A^{s}\eta \left(k\right)+\eta^{T}\left(k\right){A^{s}}^{T}\bar{P}\left(s\right)B_{1}^{s}\phi \left(y\left(k\right)\right)\\ \; & \;+\eta^{T}\left(k\right)\sum_{i=1}^{n}\alpha_{i}F_{1i}^{sT}\bar{P}\left(s\right)F_{1i}^{s}\eta \left(k\right)+\eta^{T}\left(k\right)\sum_{i=1}^{n}\alpha_{i}F_{1i}^{sT}\bar{P}\left(s\right)F_{2i}^{s}\phi \left(y\left(k\right)\right)\\ \; & \;+\phi^{T}\left(y\left(k\right)\right)B_{1}^{sT}\bar{P}\left(s\right)A^{s}\eta \left(k\right)+\phi^{T}\left(y\left(k\right)\right)B_{1}^{sT}\bar{P}\left(s\right)B_{1}^{s}\phi \left(y\left(k\right)\right)\\ \; & \;+\phi^{T}\left(y\left(k\right)\right)\sum_{i=1}^{n}\alpha_{i}F_{2i}^{sT}\bar{P}\left(s\right)F_{1i}^{s}\eta \left(k\right)+\phi^{T}\left(y\left(k\right)\right)\sum_{i=1}^{n}\alpha_{i}F_{2i}^{sT}\bar{P}\left(s\right)F_{2i}^{s}\phi \left(y\left(k\right)\right)\\ \; & \;-\eta^{T}\left(k\right)P\left(s\right)\eta \left(k\right). \end{array}$$

Denoting $\xi \left(k\right)={\left[\eta^{T}\left(k\right)\;\phi^{T}\left(y\left(k\right)\right)\right]}^{T}$, and Combining (13), then we have

(28) $$\Delta V\left(\eta \left(k\right),s\right)\leq \xi^{T}\left(k\right)\Lambda^{s}\xi \left(k\right)$$

where

(29) $$\begin{array}{cc} \Lambda^{s} & =\left[\begin{array}{cc} \Lambda_{11}^{s} & *\\ \Lambda_{21}^{s} & \Lambda_{22}^{s} \end{array}\right] \end{array}$$

and

(30) $$\begin{array}{cc} \Lambda_{11}^{s} & ={A^{s}}^{T}\bar{P}\left(s\right)A^{s}+\sum_{i=1}^{n}\alpha_{i}F_{1i}^{sT}\bar{P}\left(s\right)F_{1i}^{s}-P\left(s\right)-{\tilde{C}}^{T}{\bar{K}}_{1}^{T}{\bar{K}}_{2}\tilde{C}\\ \Lambda_{21}^{s} & =B_{1}^{sT}\bar{P}\left(s\right)A^{s}+\sum_{i=1}^{n}\alpha_{i}F_{2i}^{sT}\bar{P}\left(s\right)F_{1i}^{s}+0.5\left({\bar{K}}_{1}+{\bar{K}}_{2}\right)\tilde{C}\\ \Lambda_{22}^{s} & =\sum_{i=1}^{n}\alpha_{i}F_{2i}^{sT}\bar{P}\left(s\right)F_{2i}^{s}-I. \end{array}$$

Subsequently, using Schur complement to (23), it is obvious that matrix $\Lambda^{s}<0$, thus

(31) $$\begin{array}{c} \Delta V\left(\eta \left(k\right),s\right)\leq \xi^{T}\left(k\right)\Lambda^{s}\xi \left(k\right)\leq -\min\left\{\lambda_{\min}\left(-\Lambda^{s}\right)|s=1,2,\cdots ,n_{0}\right\}\xi^{T}\left(k\right)\xi \left(k\right)\leq -\sigma \xi^{T}\left(k\right)\xi \left(k\right) \end{array}$$

where $0<\sigma \leq \min\left\{\lambda_{\min}\left(-\Lambda^{s}\right)|\;s=1,2,\cdots ,n_{0}\right\}.$

Considering continuity, one can find a constant satisfying $0<\partial <v,v=\max\left\{\lambda_{\max}\left(P\left(s\right)\right)|s=1,2,\cdots ,n_{0}\right\}.$ Such that

(32) $$V\left(\eta \left(k\right),s\right)\leq v\xi^{T}\left(k\right)\xi \left(k\right).$$

The formulas (31) and (32) yield that

(33) $$\Delta V\left(\eta \left(k\right),s\right)\leq -\frac{\sigma }{v}V\left(\eta \left(k\right),s\right)=\lambda -\psi V\left(\eta \left(k\right),s\right)$$

where $0<\psi =\frac{\sigma }{v}<1.$

On the other hand,

(34) $$\mu {∥\eta (k)∥}^{2}\leq V\left(\eta \left(k\right),s\right)\leq v{∥\eta (k)∥}^{2}\mu =\min\left\{\lambda_{\min}\left(P\left(s\right)\right)|s=1,2,\cdots ,n_{0}\right\}.$$

Taking Lemma 1 into consideration, and selecting λ=0, it can be derived that

(35) $$E\left\{{∥\eta (k)∥}^{2}\right\}\leq \frac{v}{\mu }{\left(1-\psi \right)}^{k}E\left\{{∥\eta (0)∥}^{2}\right\}.$$

Consequently, we can conclude from Theorem 1 that the augmented system (8) is exponentially stable in the mean square sense.

Define the following performance functions

(36) $$J=E\left\{V(k)\right\}-E\left\{\sum_{i=0}^{k-1}{\bar{w}}^{T}\left(i\right)\bar{w}\left(i\right)\right\}.$$

Under the zero-initial condition,

(37) $$J=E\left\{V(k)\right\}-E\left\{V(0)\right\}-E\left\{\sum_{i=0}^{k-1}{\bar{w}}^{T}\left(i\right)\bar{w}\left(i\right)\right\}=\sum_{i=0}^{k-1}\left(\Delta V\left(i\right)-{\bar{w}}^{T}\left(i\right)\bar{w}\left(i\right)\right).$$

One can obtain immediately that

(38) $$\begin{array}{cc} \Delta V(k) & \leq \xi^{T}\left(k\right)\Lambda^{s}\xi \left(k\right)+2{\bar{w}}^{T}\left(k\right)B_{2}^{sT}\bar{P}\left(s\right)A^{s}\eta \left(k\right)\\ \; & \;+2{\bar{w}}^{T}\left(k\right)B_{2}^{sT}\bar{P}\left(s\right)B_{1}^{s}\phi \left(y\left(k\right)\right)+{\bar{w}}^{T}\left(k\right)B_{2}^{sT}\bar{P}\left(s\right)B_{2}^{s}\bar{w}\left(k\right). \end{array}$$

From (37) and (38), it follows that

(39) $$J=\sum_{i=0}^{k-1}\left(\Delta V\left(i\right)-{\bar{w}}^{T}\left(i\right)\bar{w}\left(i\right)\right)\leq \sum_{i=0}^{k-1}\left(\left[\begin{array}{cc} \xi^{T}\left(i\right) & {\bar{w}}^{T}\left(i\right) \end{array}\right]\left[\begin{array}{cc} \Lambda^{s} & *\\ \Omega_{21}^{s} & \Omega_{22}^{s} \end{array}\right]\left[\begin{array}{c} \xi (i)\\ \bar{w}\left(i\right) \end{array}\right]\right)$$

where

(40) $$\Omega_{21}^{s}=\left[\begin{array}{cc} B_{2}^{sT}\bar{P}\left(s\right)A^{s} & B_{2}^{sT}\bar{P}\left(s\right)B_{1}^{s} \end{array}\right],\;\Omega_{22}^{s}=\left[\begin{array}{cc} B_{2}^{sT}\bar{P}\left(s\right)B_{2}^{s} & -I \end{array}\right]$$

and $\Lambda^{s}$ has been defined in (29).

Applying Schur complement to (23), it guarantees J<0, then

(41) $$\begin{array}{c} E\left\{\eta^{T}\left(k\right)P\left(s\right)\eta \left(k\right)\right\}<\sum_{i=0}^{k-1}{\bar{w}}^{T}\left(i\right)\bar{w}\left(i\right). \end{array}$$

Meanwhile, by using Schur complement to (24), we obtain

(42) $$M^{sT}M^{s}<\gamma^{2}P\left(s\right).$$

By noting k>0, it is easy to be found that

(43) $$\begin{array}{ccc} E\left\{{\tilde{z}}^{T}\left(k\right)\tilde{z}\left(k\right)\right\} & = & \eta^{T}\left(k\right)M^{sT}M^{s}\eta \left(k\right)\\ & < & \gamma^{2}\eta^{T}\left(k\right)P\left(s\right)\eta \left(k\right)=\gamma^{2}E\left\{\eta^{T}\left(k\right)P\left(s\right)\eta \left(k\right)\right\}\\ & < & \gamma^{2}\sum_{i=0}^{k-1}{\bar{w}}^{T}\left(i\right)\bar{w}\left(i\right)\leq \gamma^{2}\sum_{i=0}^{\infty }{\bar{w}}^{T}\left(i\right)\bar{w}\left(i\right). \end{array}$$

According to (43), we have

(44) $$\underset{k}{\sup}\left\{E\left\{e^{T}\left(k\right)e\left(k\right)\right\}\right\}<\gamma^{2}\sum_{i=0}^{\infty }{\bar{w}}^{T}\left(i\right)\bar{w}\left(i\right).$$

Hence,

(45) $$E\left\{{∥e(k)∥}_{\infty }^{2}\right\}<\gamma^{2}{∥\bar{w}\left(k\right)∥}_{2}^{2}.$$

Consequently, the proof of Theorem 1 is completed. □

## 4. Distributed $l_{2}$-$l_{\infty }$ Filter Design

In this section, based on these established conditions, the design method of full- and reduced-order estimators is obtained.

**Theorem** **2.** *For given a prescribed disturbance attenuation level γ>0. System (8) is exponentially mean-square stable with energy-to-peak performance constraint, if there exist matrices $P_{2}\left(s\right)$, $V_{1}^{s}$, $V_{2}^{s}=diag_{n}\left\{S_{i}^{s}\right\}$, $V_{3}^{s}$, ${\bar{L}}_{f}^{s}=diag_{n}\left\{L_{i}^{s}\right\}$, $P_{1}\left(s\right)>0,P_{3}\left(s\right)>0$, and two families of matrices ${\bar{W}}_{f}^{s}$, ${\bar{H}}_{f}^{s}$ satisfying the constraints*

(46) $${\bar{W}}_{f}^{s}\in W_{n_{x}\times n_{x}},{\bar{H}}_{f}^{s}\in W_{n_{x}\times n_{y}},s=1,2,\cdots ,n_{0}$$

*and the following set of LMIs*

(47) $$\left[\begin{array}{cccccccc} \Theta_{11}^{s} & * & * & * & * & * & * & *\\ \Theta_{21}^{s} & \Theta_{22}^{s} & * & * & * & * & * & *\\ \Theta_{31}^{s} & 0 & -I & * & * & * & * & *\\ 0 & 0 & 0 & -I & * & * & * & *\\ 0 & 0 & 0 & 0 & -I & * & * & *\\ \Theta_{61}^{s} & \Theta_{62}^{s} & \Theta_{63}^{s} & \Theta_{64}^{s} & \Theta_{65}^{s} & \Theta_{66}^{s} & * & *\\ \Theta_{71}^{s} & \Theta_{72}^{s} & \Theta_{73}^{s} & \Theta_{74}^{s} & \Theta_{75}^{s} & \Theta_{76}^{s} & \Theta_{77}^{s} & *\\ \Theta_{81}^{s} & 0 & \Theta_{83}^{s} & 0 & 0 & 0 & 0 & \Theta_{88}^{s} \end{array}\right]<0$$

(48) $$\left[\begin{array}{ccc} -P_{1}\left(s\right) & * & *\\ -P_{2}\left(s\right) & -P_{3}\left(s\right) & *\\ \bar{M} & -{\bar{L}}^{s} & -\gamma^{2}I \end{array}\right]<0$$

*where*

(49) $$\begin{array}{ccc} \Theta_{11}^{s} & = & -P_{1}\left(s\right)-{\bar{C}}^{T}{\bar{K}}_{1}^{T}{\bar{K}}_{2}\bar{C},\;\Theta_{21}^{s}=-P_{2}\left(s\right),\;\Theta_{22}^{s}=-P_{3}\left(s\right),\;\Theta_{31}^{s}=0.5\left({\bar{K}}_{1}+{\bar{K}}_{2}\right)\bar{C},\\ \Theta_{61}^{s} & = & V_{1}^{sT}\bar{A}+E{\bar{H}}_{f}^{s}{\bar{C}}_{1-\beta },\;\Theta_{62}^{s}=E{\bar{W}}_{f}^{s},\;\Theta_{63}^{s}=E{\bar{H}}_{f}^{s}\bar{\beta },\;\Theta_{64}^{s}=V_{1}^{sT}\bar{B},\;\Theta_{65}^{s}=E{\bar{H}}_{f}^{s}\bar{D},\\ \Theta_{66}^{s} & = & {\bar{P}}_{1}\left(s\right)-V_{1}^{s}-V_{1}^{sT},\;\Theta_{71}^{s}=V_{3}^{sT}\bar{A}+{\bar{H}}_{f}^{s}{\bar{C}}_{1-\beta },\;\Theta_{72}^{s}={\bar{W}}_{f}^{s},\;\Theta_{73}^{s}={\bar{H}}_{f}^{s}\bar{\beta },\\ \Theta_{74}^{s} & = & V_{3}^{sT}\bar{B},\;\Theta_{75}^{s}={\bar{H}}_{f}^{s}\bar{D},\;\Theta_{76}^{s}={\bar{P}}_{2}\left(s\right)-V_{3}^{sT}-V_{2}^{sT}E^{T},\;\Theta_{77}^{s}={\bar{P}}_{3}\left(s\right)-V_{2}^{s}-V_{2}^{sT},\\ \Theta_{81}^{s} & = & vec_{n}\left\{\left[\begin{array}{c} -\sqrt{\alpha_{i}}E{\bar{H}}_{f}^{s}{\bar{C}}_{n}^{i}\\ -\sqrt{\alpha_{i}}{\bar{H}}_{f}^{s}{\bar{C}}_{n}^{i} \end{array}\right]\right\},\;\Theta_{83}^{s}=vec_{n}\left\{\left[\begin{array}{c} \sqrt{\alpha_{i}}E{\bar{H}}_{f}^{s}{\bar{E}}_{n}^{i}\\ \sqrt{\alpha_{i}}{\bar{H}}_{f}^{s}{\bar{E}}_{n}^{i} \end{array}\right]\right\},\\ \Theta_{88}^{s} & = & diag_{n}\left\{\left[\begin{array}{cc} {\bar{P}}_{1}\left(s\right)-V_{1}^{s}-V_{1}^{sT} & *\\ {\bar{P}}_{2}\left(s\right)-V_{3}^{sT}-V_{2}^{sT}E^{T} & {\bar{P}}_{3}\left(s\right)-V_{2}^{s}-V_{2}^{sT} \end{array}\right]\right\}. \end{array}$$

*The filter parameters can be expressed as follows:*

(50) $${\bar{W}}^{s}=V_{2}^{sT^{-1}}{\bar{W}}_{f}^{s},\;{\bar{H}}^{s}=V_{2}^{sT^{-1}}{\bar{H}}_{f}^{s},\;{\bar{L}}^{s}={\bar{L}}_{f}^{s}.$$

**Proof** **of Theorem 2.** By choosing

(51) $$G^{s}=\left[\begin{array}{cc} V_{1}^{s} & V_{3}^{s}{Q^{s}}^{{}^{-1}}F^{s}\\ Q^{s}E^{T} & F^{s} \end{array}\right]$$

where

(52) $$E=\left[\begin{array}{c} I_{nl*nl}\\ 0_{n(m-l)*nl} \end{array}\right],\;V_{1}^{s}\in R^{nm*nm},\;V_{3}^{s}\in R^{nm*nl},\;Q^{s}\in R^{nl*nl},\;F^{s}\in R^{nl*nl}.$$

In light of Lemma 2, it can be derived that

(53) $$\left[\begin{array}{ccccc} \Phi_{11}^{s} & * & * & * & *\\ \Phi_{21}^{s} & -I & * & * & *\\ 0 & 0 & -I & * & *\\ \Phi_{41}^{s} & \Phi_{42}^{s} & \Phi_{43}^{s} & \Phi_{44}^{s} & *\\ \Phi_{51}^{s} & \Phi_{52}^{s} & 0 & 0 & \Phi_{55}^{s} \end{array}\right]<0$$

where

(54) $$\begin{array}{ccc} \Phi_{11}^{s} & = & -P\left(s\right)-{\tilde{C}}^{T}{\bar{K}}_{1}^{T}{\bar{K}}_{2}\tilde{C},\;\Phi_{21}^{s}=0.5\left({\bar{K}}_{1}+{\bar{K}}_{2}\right)\tilde{C},\\ \Phi_{41}^{s} & = & G^{sT}A^{s},\;\Phi_{42}^{s}=G^{sT}B_{1}^{s},\;\Phi_{43}^{s}=G^{sT}B_{2}^{s},\\ \Phi_{44}^{s} & = & \bar{P}\left(s\right)-G^{s}-G^{sT},\;\Phi_{51}^{s}=vec_{n}\left\{\sqrt{\alpha_{i}}G^{T}\left(s\right)F_{1i}^{s}\right\},\\ \Phi_{52}^{s} & = & vec_{n}\left\{\sqrt{\alpha_{i}}G^{T}\left(s\right)F_{2i}^{s}\right\},\;\Phi_{55}^{s}=diag_{n}\left\{\bar{P}\left(s\right)-G^{s}-G^{sT}\right\}. \end{array}$$

Denote

(55) $$\begin{array}{cc} V_{2}^{s} & ={Q^{s}}^{T}F^{s^{-1}}Q^{s},\;J^{s}=\left[\begin{array}{cc} I & 0\\ 0 & F^{s^{-1}}Q^{s} \end{array}\right],\\ J^{sT}P\left(s\right)J^{s} & =\left[\begin{array}{cc} P_{1}\left(s\right) & P_{2}^{T}\left(s\right)\\ P_{2}\left(s\right) & P_{3}\left(s\right) \end{array}\right],\;J^{sT}\bar{P}\left(s\right)J^{s}=\left[\begin{array}{cc} {\bar{P}}_{1}\left(s\right) & {\bar{P}}_{2}^{T}\left(s\right)\\ {\bar{P}}_{2}\left(s\right) & {\bar{P}}_{3}\left(s\right) \end{array}\right]. \end{array}$$

Pre- and post-multiplying (24) and (53) by $T_{1}^{s}=diag\left\{J^{s},I,I,J^{s},J^{s}\right\}$ and $T_{2}^{s}=diag\left\{J^{s},I\right\}$, respectively. Further define ${\bar{W}}_{f}^{s}=Q^{sT}{\bar{W}}^{s}{F^{s}}^{{}^{-1}}Q^{s}$, ${\bar{H}}_{f}^{s}=Q^{sT}{\bar{H}}^{s}$, ${\bar{L}}_{f}^{s}={\bar{L}}^{s}{F^{s}}^{{}^{-1}}Q^{s}$, $V_{2}^{s}=Q^{sT}{F^{s}}^{{}^{-1}}Q^{s}$, then the inequality (47) and (48) can be obtained readily.

Now, denoting the filter transfer function from $\tilde{y}\left(k\right)$ to $\hat{x}\left(k\right)$ by

(56) $$T_{z_{F}y}\left(z\right)={\bar{L}}^{s}{\left(zI-{\bar{W}}^{s}\right)}^{-1}{\bar{H}}^{s}.$$

Considering

(57) $${\bar{W}}^{s}=Q^{sT^{-1}}{\bar{W}}_{f}^{s}Q^{s^{-1}}F^{s},\;{\bar{H}}^{s}=Q^{sT^{-1}}{\bar{H}}_{f}^{s},\;{\bar{L}}^{s}={\bar{L}}_{f}^{s}Q^{s^{-1}}F^{s}$$

we have

(58) $$\begin{array}{cc} T_{z_{F}y}\left(z\right) & ={\bar{L}}_{f}^{s}Q^{s^{-1}}F^{s}{\left(zI-Q^{sT^{-1}}{\bar{W}}_{f}^{s}Q^{s^{-1}}F^{s}\right)}^{-1}Q^{sT^{-1}}{\bar{H}}_{f}^{s}\\ \; & ={\bar{L}}_{f}^{s}{\left(zQ^{s^{-1}}F^{s}-Q^{sT^{-1}}{\bar{W}}_{f}^{s}\right)}^{-1}Q^{sT^{-1}}{\bar{H}}_{f}^{s}\\ \; & ={\bar{L}}_{f}^{s}{\left(zI-{Q^{s}}^{{}^{-1}}F^{s}{Q^{sT}}^{{}^{-1}}{\bar{W}}_{f}^{s}\right)}^{-1}Q^{s^{-1}}F^{s}Q^{sT^{-1}}{\bar{H}}_{f}^{s}\\ \; & ={\bar{L}}_{f}^{s}{\left(zI-V_{2}^{s^{-1}}{\bar{W}}_{f}^{s}\right)}^{-1}V_{2}^{s^{-1}}{\bar{H}}_{f}^{s}. \end{array}$$

Then, the distributed full- and reduced-order filter parameters are given by

(59) $${\bar{W}}^{s}=V_{2}^{s^{-1}}{\bar{W}}_{f}^{s},\;{\bar{H}}^{s}=V_{2}^{s^{-1}}{\bar{H}}_{f}^{s},\;{\bar{L}}^{s}={\bar{L}}_{f}^{s}.$$

Moreover, it follows from Lemma 3 that ${\bar{W}}^{s}\in W_{n_{x}\times n_{x}}$, ${\bar{H}}^{s}\in W_{n_{x}\times n_{y}}$. The proof of Theorem 2 is now complete. □

**Remark** **2.** *Eliminating the coupling term between Lyapunov matrix P(s) and system matrices by introducing additional slack variables $G^{s}$. Then, it is found that (23) and (53) are equivalent, which helps us to easily obtain the filter (5).*

**Remark** **3.** *The order of the filter is controlled by the column dimension nl of matrix E (Where n, m, l represent the number of filter nodes, the order of full- and reduced-order filter, respectively). If the column dimension number nl = nm of matrix E, that is, matrix E is a unit matrix. Then the order of each local filter node is m, which means that it is a full order filter. Similarly, if 1≤nl<nm, we will get the l-order filter, which is the reduced-order filter.*

**Remark** **4.** *In Theorem 2, the design approach of distributed l-order filters with non-Gauss interference input and deception attak is given. Then, the problem can be transformed into a feasible solution of linear matrix inequality (46)–(48), which is solved by the following convex optimization problem, where $\delta =\gamma^{2}$*

(60) $$\underset{s.t.\;\left(46)–(48\right)}{\min_{P_{1}\left(s\right),P_{2}\left(s\right),P_{3}\left(s\right),V_{1}^{s},V_{2}^{s},V_{3}^{s},{\bar{W}}_{f}^{s},{\bar{H}}_{f}^{s},{\bar{L}}_{f}^{s}}}\delta .$$

In the next section, we will validate the design scheme of distributed state estimator by a numerical simulation example.

## 5. Simulation Example

In order to illustrate the effectiveness of the proposed distributed filtering design approach in this paper, consider discrete-time system (1) with the following parameters:

$$\left\{\begin{array}{c} \begin{array}{cc} x(k+1) & =\left[\begin{array}{cc} 0 & 0.3\\ -0.5 & 0.6 \end{array}\right]x\left(k\right)+\left[\begin{array}{c} 0\\ 1 \end{array}\right]w\left(k\right)\\ z(k) & =\left[\begin{array}{cc} 0.4 & 0.1 \end{array}\right]x\left(k\right). \end{array} \end{array}\right.$$

Consider a distributed filtering network with 4 sensor nodes with the following corresponding parameters:

$$\begin{array}{ccc} C_{1} & = & \left[\begin{array}{cc} 0.1 & 0.5 \end{array}\right],\;C_{2}=\left[\begin{array}{cc} 0.1 & -0.2 \end{array}\right],\;C_{3}=\left[\begin{array}{cc} -0.5 & 0.5 \end{array}\right],\;C_{4}=\left[\begin{array}{cc} 0.1 & -0.4 \end{array}\right],\\ D_{1} & = & 0.11,\;D_{2}=0.15,\;D_{3}=0.14,\;D_{4}=0.21,\;K_{1}=0.1,\;K_{2}=-1,\;v_{i}\left(k\right)=N\left(0,\frac{1}{k^{2}}\right),\\ w(k) & = & e^{-0.2k}\sin\left(k\right),\;\phi_{i}\left(y_{i}\left(k\right)\right)=\sin\left(-0.7C_{i}x\left(k\right)\right),i=1,2,3,4. \end{array}$$

The probability of deception attacks are taken as:

$$\begin{array}{cc} \beta_{1} & =E\left\{\theta_{1}\left(k\right)\right\}=0.1,\;\beta_{2}=E\left\{\theta_{2}\left(k\right)\right\}=0.2,\\ \beta_{3} & =E\left\{\theta_{3}\left(k\right)\right\}=0.4,\;\beta_{4}=E\left\{\theta_{4}\left(k\right)\right\}=0.1. \end{array}$$

**Figure 2:**
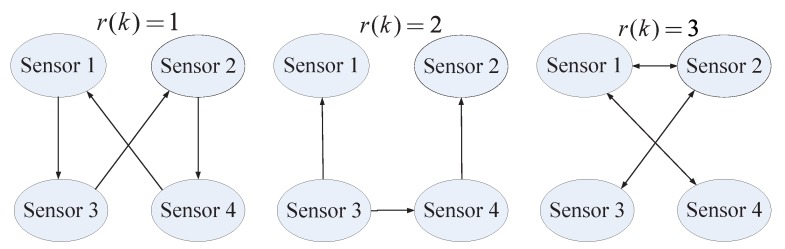
Filtering network topology.

The filtering network communication topology shown in Figure 2 is represented by three directed graphs $G=\left(V,E,A\right)$ with the set of nodes V=1,2,3,4, the set of edges $E^{r(1)}=\left\{\left(1,1),(1,4),(2,2),(2,3),(3,1),(3,3),(4,2),(4,4\right)\right\},\;E^{r(2)}=\left\{\left(1,1),(1,3),(2,2),(2,4),(3,3),(4,3),(4,4\right)\right.$, $E^{r(3)}=\left\{\left(1,1),(1,2),(1,4),(2,1),(2,2),(2,3),(3,2),(3,3),(4,4\right)\right\}$. Let the initial topology of the filtering network be $r_{0}=1$, and the filter initial states ${\hat{x}}_{i}\left(0\right)$ are all set to be zeros.

Consider the following probability transmission matrix of Markov chain:

$$\Pi =\left[\begin{array}{ccc} 0.6 & 0.2 & 0.2\\ 0.2 & 0.7 & 0.1\\ 0.1 & 0.1 & 0.8 \end{array}\right].$$

In order to obtain full-order filters, we choose order control parameter

$$E=\left[\begin{array}{cccccccc} 1 & 0 & 0 & 0 & 0 & 0 & 0 & 0\\ 0 & 1 & 0 & 0 & 0 & 0 & 0 & 0\\ 0 & 0 & 1 & 0 & 0 & 0 & 0 & 0\\ 0 & 0 & 0 & 1 & 0 & 0 & 0 & 0\\ 0 & 0 & 0 & 0 & 1 & 0 & 0 & 0\\ 0 & 0 & 0 & 0 & 0 & 1 & 0 & 0\\ 0 & 0 & 0 & 0 & 0 & 0 & 1 & 0\\ 0 & 0 & 0 & 0 & 0 & 0 & 0 & 1 \end{array}\right].$$

By solving the LMIs in Theorem 2, we solve the switching topology-dependent optimal problem (60) subject to (46)–(48), and obtain the optimal $l_{2}$-$l_{\infty }$ disturbance rejection attenuation level γ=0.2450.

Then, we consider a reduced-order case where the order control parameter

$$E=\left[\begin{array}{cccc} 1 & 0 & 0 & 0\\ 0 & 1 & 0 & 0\\ 0 & 0 & 1 & 0\\ 0 & 0 & 0 & 1\\ 0 & 0 & 0 & 0\\ 0 & 0 & 0 & 0\\ 0 & 0 & 0 & 0\\ 0 & 0 & 0 & 0 \end{array}\right].$$

Keep other parameters the same as the full-order filtering. Simultaneously, we obtain γ=0.4731 of reduced-order filter.

**Figure 3:**
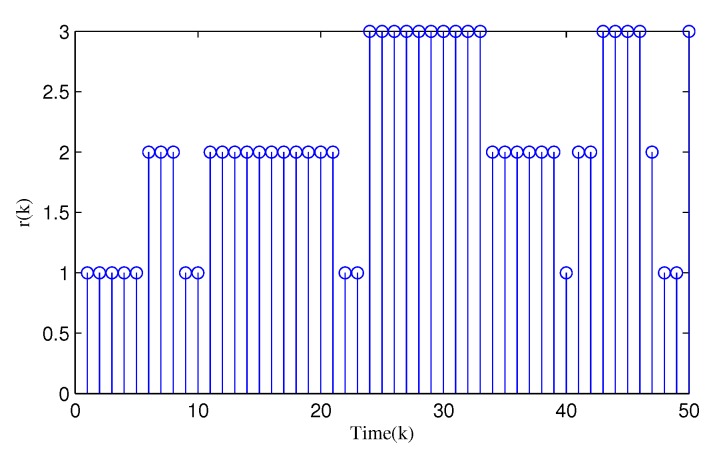
Evolution of r(k).

**Figure 4:**
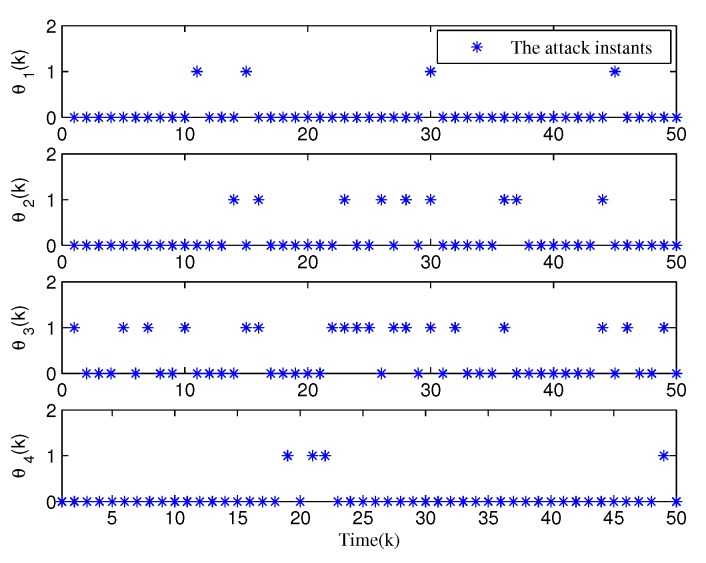
Evolution of attack variable $\theta_{i}\left(k\right),i=1,2,3,4.$

**Figure 5:**
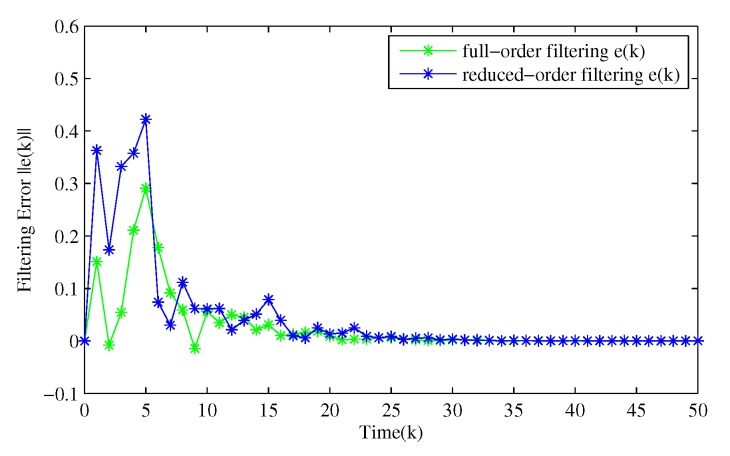
Evolution of full- and reduced-order filtering e(k).

**Figure 6:**
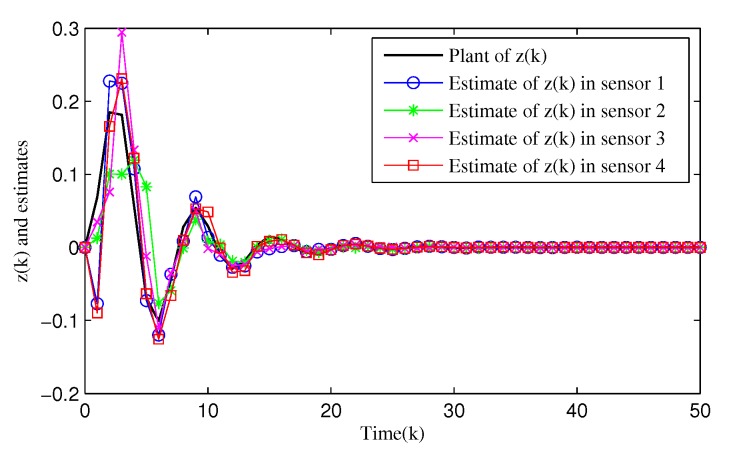
Evolution of full-order filtering ${\hat{z}}_{i}\left(k\right),i=1,2,3,4.$

**Figure 7:**
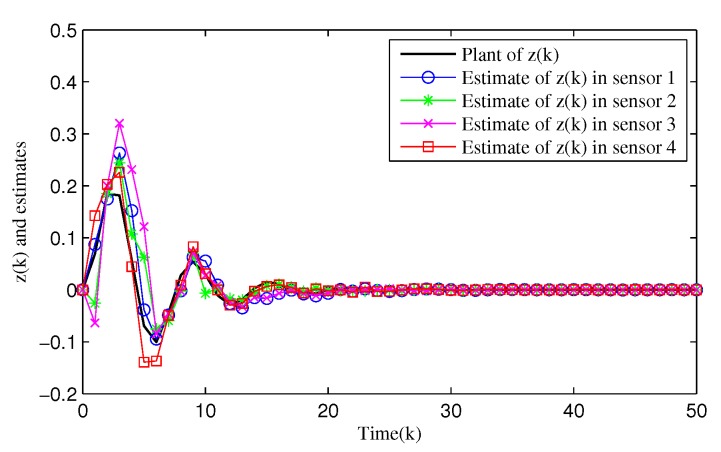
Evolution of reduced-order filtering ${\hat{z}}_{i}\left(k\right),i=1,2,3,4.$

From Figure 3, Figure 4, Figure 5, Figure 6 and Figure 7, the corresponding simulation results between the full- and reduced-order filtering can be obtained. The evolution of the Markov chain is depicted in Figure 3. The attack instants of each communication channel is shown in Figure 4, where $\theta_{i}\left(k\right)=1$ means that the local sensor node is under malicious attacks and the communication channel is injected with false information. Correspondingly, the sensor node works normally at $\theta_{i}\left(k\right)=0$.

Figure 5 shows the dynamics of the filtering error norm of full- and reduced-order estimators, where $e\left(k\right)=\sum_{i=1}^{4}∥e(i)∥$, respectively. This confirms that the designed distributed full-order and distributed reduced-order filtering are both feasible and effective. Figure 6 and Figure 7 describe in detail the evolution of *z*(*k*) and its estimation from full- and reduced-filter for each sensor node 1, 2, 3 and 4, respectively. It can be easily seen that the filter estimation error is relatively large at the beginning, and then the accuracy of the estimation is gradually improved. By comparing Figure 5, Figure 6 and Figure 7, we can observe that (i) The overall estimation error of the distributed reduced-order filter slightly higher than that of the distributed full-order filter (ii) Malicious attacks do cause filter performance degradation, whether it is a full or reduced-order filter. Specifically, although the full-order filter performs better than the reduced-order filter, the distributed reduced-order filter is easier to apply to engineering.

It should be noted that, for the problem solved in this paper, (i) External interference in sensor networks does not require known statistical properties, and (ii) The order of the distributed filter is controlled by a parameter, and distributed full-order and distributed reduced-order filters can be obtained for each node of the sensor networks, respectively. Unfortunately, the first factor prevents existing methods (Kalman filtering, extended Kalman filtering, see [11,14,19,39]) from being applied to distributed state estimation problems for sensor networked systems with unknown interference. On the other hand, the second factor is ignored by many existing $H_{\infty }$ methods, see [16,38,42,43], which often focus on designing distributed full-order filters over WSNs.

## 6. Conclusions and Future Work

In this paper, the problem of *l*-order distributed $l_{2}$-$l_{\infty }$ state estimation problem for a class of discrete time-invariant systems has been investigated. Through an adjustable parameter *E*, a distributed full-order filter and a distributed reduced-order filter can be obtained respectively. During the design of the filter, the random deception attacks, time-varying communication topology, stochastic noises are simultaneously considered to reflect more practical dynamic behavior of WSNs. By utilizing the Markovian switched Lyapunov functional method and the LMI technique, sufficient conditions on the designed distributed estimator have been obtained to ensure the prescribed energy-to-peak performance with given filter parameters. The filtering parameters can be determined and characterized with the explicit expressions by solving some LMIs. Finally, the validity of the design approach was verified by numerical simulation. In the future, we will concentrate on how to extend the achieved approachs to deal with filtering problems over WSNs with time-varying switching topologies under different communication protocols, such as round-robin protocol, stochastic communication protocol and weighted try-once-discard protocol, would be of interest.
